# Maternal health service utilization and birth outcomes among female sex workers in the Democratic Republic of the Congo, Kenya, and Nigeria: results from a cross-sectional survey

**DOI:** 10.1186/s12889-026-28825-9

**Published:** 2026-08-12

**Authors:** Isabella Danel, Regan Moss, Brian Willis, Swarna D. S. Weerasinghe, Heather Thompson, Kelsey Bricker, Patrick Ezie, Grace Kamau, Emily Perttu, Wendy L. Macias-Konstantopoulos

**Affiliations:** 1 Global Health Promise, Portland, OR USA; 2 Division of Community Health Sciences, School of Public Health, University of Illinois, Chicago, IL USA; 3 Social, Behavioral, and Population Health Sciences, Celia Scott Weatherhead School of Public Health, Tulane University, New Orleans, LA USA; 4 Department of Community Health and Epidemiology, Faculty of Medicine, Dalhousie University, Halifax, NS Canada; 5 Faculty of Medicine, McMaster University, Hamilton, ON Canada; 6 Silver Cross Hospital, Federal Capital Territory, Abuja Nigeria; 7 African Sex Workers Alliance, Nairobi, Kenya; 8 Center for Social Justice and Health Equity, Department of Emergency Medicine, Massachusetts General Hospital, Harvard Medical School, Boston, MA USA

**Keywords:** Female sex workers, Maternal health services, Birth outcomes, Antenatal care, Skilled birth attendance, Stillbirths, Perinatal mortality, Low birth weight, Alcohol use, Health equity

## Abstract

**Background:**

Information on maternal health service utilization among female sex workers (FSW) in low- and middle-income countries (LMIC) is limited, with research focusing on HIV. Despite most FSW being mothers, little is known about their childbirth care or birth outcomes. To our knowledge, this is the first multicountry study to describe adverse birth outcomes among FSW in LMIC.

**Methods:**

This cross-sectional study used a convenience sample of 853 FSW from eight cities in the Democratic Republic of the Congo (DRC), Kenya, and Nigeria to examine antenatal care (ANC) utilization, substance use during pregnancy, skilled birth attendance, and birth outcomes (stillbirth, perinatal mortality, and low birth weight). Self-reported outcomes for the five years preceding data collection were compared descriptively with national survey data for urban women to provide context.

**Results:**

Maternal health service utilization and birth outcomes varied widely by country. FSW participants in Kenya reported high ANC utilization (95.6%) and facility delivery (97.0%), comparable to urban women. ANC utilization and facility delivery were lower among FSW participants in the DRC (ANC: 49.0%; facility delivery: 68.0%) and Nigeria (ANC: 37.5%; facility delivery: 26.1%) compared to urban women. Cost and stigma were the main barriers. Alcohol use during pregnancy was common in all countries. Stillbirth rates among participating FSW were very high (113 per 1000 births in the DRC [95% CI 85–146], 49 in Kenya [95% CI 30–75], and 85 in Nigeria [95% CI 63–111]), as was perinatal mortality. Low birth weight prevalence was high in the two countries with sufficient birthweight data (DRC: 17.1%; Kenya: 9.7%).

**Conclusions:**

In this study sample, adverse birth outcomes—high stillbirth and perinatal mortality and low birth weight—may reflect the combined effects of limited access to quality maternal health services (MHS) and broader social, economic and health vulnerabilities documented among FSW populations. Kenya’s subsidized maternal healthcare system may contribute to higher MHS utilization and better birth outcomes observed in Kenya. Improving equity in maternal and newborn health for FSW requires increasing access to quality MHS and addressing underlying social determinants of health. FSW should be recognized as a high-risk population requiring targeted maternal health interventions.

**Supplementary Information:**

The online version contains supplementary material available at https://doi.org/10.1186/s12889-026-28825-9.

## Background

Most female sex workers (FSW) in low- and middle-income countries (LMIC) become pregnant and are mothers [1, 2]. However, there is limited information about their maternal health and birth outcomes [3]. Most research and health programs targeting FSW focus on HIV prevention and prevention of mother to child transmission. Existing research on maternal health service utilization among FSW in LMIC is largely limited to antenatal care with minimal data on childbirth experiences and health behaviors during pregnancy, and virtually no data on adverse birth outcomes [4].

FSW experience unique maternal health risks due to occupational exposures, including sexually transmitted infections and client-perpetrated violence. These risks are compounded by broader structural and social determinants of health, including poverty, food insecurity, unstable housing, limited education, stigma, and discrimination. FSW are also at heightened risk for intimate partner violence which is associated with poor maternal and perinatal health outcomes, including stillbirth [5]. A lack of legal protection further exposes FSW to theft, violence, and abuse from clients. Together, these occupational, social, and legal factors create a context of structural vulnerability that may increase the risk of adverse maternal and newborn outcomes.

Access to quality maternal healthcare, including antenatal care (ANC), skilled birth attendance and postpartum care, is known to improve maternal and perinatal health outcomes [6, 7]. However, ANC utilization among FSW is variable and influenced by barriers including stigma (external and internalized), discrimination and cost [4]. In many countries, sex work is criminalized and highly stigmatized. FSW face discrimination, limiting access to healthcare and increasing vulnerability to adverse outcomes [8]. Stigma related to sexually transmitted infections and HIV further complicates care-seeking [9]. As a result, many FSW avoid disclosing their occupation, which can hinder the provision of appropriate care [10]. Additionally, the cost of maternal health services (MHS) presents a significant barrier, for many low-income women.

The number of visits and the quality of ANC influence health outcomes, yet few studies on ANC use among FSW consider these factors. Similarly, despite the critical role of skilled birth attendance, and the barriers FSW face in accessing quality care, there is limited information on their childbirth experiences and birth outcomes. We identified only one study from sub-Saharan Africa reporting on skilled birth attendance and no studies on birth outcomes among FSW in LMICs, with just one study from a high-income country [11, 12].

A recent multi-country study using the Community Knowledge Approach retrospectively identified 1632 deaths among FSW, a predominantly young population of women of reproductive age, in the DRC, Kenya, and Nigeria over a five-year period (2014–2019); maternal causes accounted for 59% to 88% of all reported deaths, a proportion of all-cause mortality rather than a conventional birth-based maternal mortality ratio [13]. The primary causes of maternal death included abortion, hemorrhage and suicide.

These countries represent diverse cultural, health system and policy contexts. Access to MHS varies substantially: Kenya has implemented national programs to reduce financial barriers to care, while the DRC is in the process of implementing similar reforms. All three countries have HIV programs targeting FSW, but MHS are delivered largely through the general health system and are not tailored to their needs. Legal and social contexts also differ but are uniformly restrictive. Sex work is illegal in the northern states of Nigeria. In southern Nigeria, Kenya, and the DRC, the exchange of sex between consenting adults is generally not prohibited; however, related activities such as pimping, operating a brothel, procuring, soliciting, and loitering may be criminalized, leaving FSW vulnerable to police harassment, extortion, and violence. Stigma is pervasive across all settings. Together, these differences in health systems, legal environments, and social conditions may shape FSWs’ access to care and maternal and perinatal health outcomes.

This study addresses critical gaps in knowledge by assessing MHS utilization and adverse birth outcomes among FSW in the DRC, Kenya, and Nigeria, where maternal causes account for a high proportion of deaths among FSW [13]. We examine barriers to care and key indicators across the continuum of maternal health, including antenatal care, substance use during pregnancy, skilled birth attendance, and birth outcomes, specifically low birth weight (LBW), stillbirth, and perinatal mortality. Outcomes observed among FSW are presented alongside those of urban women from nationally representative health surveys to provide public health context and illustrate disparities, rather than to support population-level inference. The study was descriptive in nature and was not designed to assess causal relationships.

## Methods

### Study design and participants

This study is part of a broader investigation into the health and nutrition of FSW and their children in urban areas of the DRC, Kenya, and Nigeria. A cross-sectional survey with retrospective self-reported data was conducted between February 2022 and February 2023 across eight cities. Country site selection was based on criteria including a large FSW population, a high maternal mortality rate, a high HIV infection rate among FSWs, and the presence of local sex worker organizations (SWOs) and non-governmental organizations (NGOs) working with FSW populations. Within each country, cities were selected based on the recommendations and expertise of local SWOs and NGOs and prior successful collaboration conducting research with FSW populations in these locations [13]. Selection considered the presence of sizable FSW populations, logistical feasibility, and safety for study personnel.

The study design included three cities per country, enrolling 100 FSW per city. Due to social unrest, data collection in Goma (DRC) was halted, and the Kinshasa sample was increased to 150 to help compensate. Eligibility criteria included: 18 years or older, engaged in full-time sex work for the past three years, and the biological mother of at least one child aged five years or younger. Full-time sex work was self-defined by participants. The third criterion ensured adequate data on maternal health experiences, birth outcomes, and child health.

Participants were recruited by local sex worker led organizations in Kenya and Nigeria and by non-governmental organizations providing HIV services to FSW in the DRC. These organizations regularly did outreach and were familiar with local spots frequented by FSW. A convenience sample of FSW was recruited from a variety of venues including hot spots, bars, brothels, and streets. Because FSW are a hard-to-reach population, no sampling frame was available, precluding probability-based sampling. Consequently, findings are not intended to be population-representative but rather to characterize maternal health experiences and outcomes among participating FSW across diverse urban settings.

Local staff visited different venues every night and shared information about the study with FSW. They would screen potential participants at the hotspot and invite eligible FSW to the interview location the next day where they received additional information about the study. Childcare was provided to facilitate participation. Participants received modest non-coercive compensation that included a meal and a small cash reimbursement for time and transport.

### Data collection and study instrument

The survey was conducted by staff members of local organizations to help facilitate interviewer-participant trust. Interviews were conducted in safe, participant-friendly locations identified by the local organizations. To promote consistency across sites all interviewers were trained by the lead researcher (BW) during an approximately half-day training session covering review of each questionnaire item, standardized interviewing procedures, and administration of sensitive questions. At each site, a separate staff member was assigned to administer each of the four questionnaire modules throughout data collection. This included a dedicated interviewer for the maternal health module used in the present analyses, further promoting consistency. The lead researcher remained onsite throughout data collection to answer questions and monitor adherence to study procedures. Completed questionnaires were reviewed for missing responses and legibility before the participant left the venue.

In-person interviews were conducted using a structured paper-based questionnaire. Interviews in Kenya and Nigeria were conducted in English. In the DRC, the questionnaire was translated into French and trained bilingual staff members from local partner organizations conducted interviews in Swahili (Bukavu) or Lingala (Kinshasa), translating questions orally. Formal back-translation was not performed. Completed questionnaires were translated into English by professional translators prior to data entry.

To protect confidentiality of participants, no personally identifiable information was collected. The interviewers in each country read and reviewed the study information sheet and the informed consent form in the local language with potential participants in coordination with the lead investigator. These documents included information about the sensitive nature, purpose, and duration of the study, the potential harms and benefits of participation, details about compensation, and their rights as voluntary study participants to ask for clarifications, decline to answer questions, request breaks, withdraw from the study at any time, and seek mental health counseling through the local partner organizations. Following an interactive assessment of comprehension with ample opportunity for clarification and discussion, those who agreed to participate marked their consent with an “X” or checkmark to accommodate literacy limitations.

The questionnaire included four modules: food insecurity, depression, FSW deaths, and maternal and child health. This paper focuses on maternal and child health questions related to MHS utilization, substance use, and birth outcomes, including LBW, stillbirths, and perinatal mortality. (The questionnaire module used for this study is provided in Supplementary Material 1). Other topics—such as contraception and abortion, HIV, and child and FSW mortality will be analyzed in separate papers. Many maternal and child health questions were adapted from the Demographic and Health Survey (DHS) to enable comparisons with national survey data. Questions related to ANC and delivery care were based on DHS and referred to the most recent pregnancy resulting in a live birth. Participants reporting no ANC or facility-based delivery were asked to identify barriers from a structured list, with multiple responses allowed and an open-ended “other” option permitted. Substance use questions referred to this same pregnancy and assessed the type and quantity of alcohol and/or drug use. For participants who had experienced a child death, questions were also adapted from DHS and included the date of death and date of birth or age at death. Unlike the DHS, a complete pregnancy history was not collected. Instead, participants reported the number of live births, stillbirths, abortions, and miscarriages within the past five years. As a result, analyses were limited to aggregate counts of births and adverse outcomes and could not assess risk factors at the individual pregnancy level.

### Data analysis and comparisons with population-based surveys

Descriptive analyses were performed using SAS OnDemand for Academics and Excel, with text data analyzed using RapidMiner. Responses to questions on pregnancies within the past five years, including the number of live births and stillbirths, were used to calculate the mean number of live births per woman and stillbirth rates within the study sample. Stillbirths were defined as fetal deaths at six months’ gestation or later. A month-based definition was used because gestational age in these settings is commonly reported in months rather than weeks, reflecting limited access to early ultrasound and imprecise pregnancy dating. The stillbirth rate was calculated as the number of stillbirths per 1,000 total births (live births + stillbirths) over the five-year period.

Perinatal deaths were defined as the sum of stillbirths and early neonatal deaths (0–6 days), with the perinatal mortality rate (PMR) calculated per 1,000 total births (live births + stillbirths). Date of death and date of birth or age at death were used to identify early neonatal deaths. Early neonatal deaths were defined as deaths occurring within 0–6 days among live births reported during the five-year recall period. Because classification of stillbirths and neonatal deaths relied on retrospective maternal report, some misclassification between miscarriage, stillbirth, and early neonatal death is possible.

Exact 95% confidence intervals were calculated for stillbirth and perinatal mortality rates to describe sampling variability within the study sample. However, because the study used a convenience sample, these intervals do not account for potential selection bias and should not be interpreted as population-representative estimates for the broader FSW population.

Responses to questions about the most recent live birth formed the basis for analyses of ANC, substance use during pregnancy, care at delivery, and LBW. The denominator for these indicators is slightly smaller than the total interviewed because it includes only women who had a live birth in the five years before the survey.

To provide context for interpreting the findings, results were compared descriptively with those of urban women from the most recent population-based surveys in each country. Formal statistical testing was not performed because differences in sampling design, target populations, recall periods, and indicator definitions could lead to misleading inferences [14–16]. Urban populations were selected as the reference group to better approximate the service environment of FSW in this study. While additional disaggregation by both urban residence and socioeconomic status would have been ideal, this information is not available in the population-based survey reports. The surveys varied in recall periods (two to five years) and denominators for some variables (e.g., all births vs. most recent births). Key differences between the FSW survey and population-based surveys are summarized in Supplementary Material 2.

### Comparison of FSW stillbirth rates with global burden of diseases study estimates

DHS surveys are known to underestimate stillbirth rates and define stillbirths as fetal deaths at 28 weeks or 7 months gestation or later [14–17]. To address this limitation, we also compared our findings with national estimates from the Global Burden of Disease (GBD) 2021 Study, which defines stillbirths as fetal deaths at 20 weeks or later [18]. As a result, GBD stillbirth rate estimates are higher than those from DHS, providing a more conservative benchmark for comparison. GBD estimates are based on data from multiple sources, including surveys, published studies, and vital registration systems.

## Results

### FSW Characteristics

A total of 853 FSW participated: 302 in Kenya, 250 in the DRC, and 301 in Nigeria (Table 1). Approximately 100 FSW participated in each city, except Kinshasa which had 150. Mean age varied by country: 24.0 in the DRC, 28.3 in Nigeria, and 30.2 years in Kenya. Most participants were single or never married, with none married in the DRC and Nigeria and only two in Kenya.

**Table 1:** Characteristics of FSW participants

Characteristics	DRC Jan 27-Feb28 2023 *n* (%) (*N* = 250)	Kenya Feb 10-Mar 8 2022 *n* (%) (*N* = 302)	Nigeria Mar 22-Apr 28 2022 *n* (%) (*N* = 301)
City			
Abuja	-	-	100 (33.2)
Bukavu	100 (40.0)	-	-
Calabar	-	-	101 (33.6)
Kinshasa	150 (60.0)	-	-
Kisumu	-	100 (33.1)	-
Lagos	-	-	100 (33.2)
Mombasa	-	99 (32.8)	-
Nairobi	-	103 (34.1)	-
Age			
18-24	140 (56.0)	50 (16.6)	90 (29.9)
25-29	85 (34.0)	97 (32.1	94 (31.2)
30-39	25 (10.0)	136 (45.0)	106 (35.2)
40+	0	19 (6.3)	11 (3.7)
Mean age±SD	24.0±3.9	30.2±5.8	28.3±6.1
Marital Status			
Single/never married	245 (98.0)	217 (71.9)	221 (73.4)
Married	0	2 (0.7)	0
Divorced/Separated	5 (2.0)	64 (21.2)	44 (14.6)
Widowed	0	14 (4.6)	34 (11.3)
No response	0	5 (1.7)	2 (0.7)
Education level			
No education	52 (20.8)	2 (0.7)	17 (5.6)
Primary	80 (31.7)	126 (41.7)	97 (32.2)
Secondary	95 (38.0)	142 (47.0)	177 (58.8)
Advanced degree	23 (9.2)	32 (10.6)	9 (3.0)
No response	0	0	1 (0.3)
Current HIV status			
Negative	120 (48.0)	229 (75.8)	238 (79.1)
Positive	14 (5.6)	51 (16.9)	25 (8.3)
Don’t know	115 (46.0)	17 (5.6)	31 (10.3)
No response	1 (0.4)	5 (1.7)	7 (2.3)

Educational attainment differed across countries: 47.2% of FSW participants in the DRC, 57.6% in Kenya, and 61.8% in Nigeria had completed some secondary education or higher. The proportion with no formal education was highest in the DRC (20.8%), lowest in Kenya (< 1%), and 5.6% in Nigeria.

HIV prevalence also varied: Kenya had the highest proportion of FSW reporting an HIV-positive status (16.9%) and the lowest proportion unaware of their HIV status (5.6%). In the DRC 5.6% of FSW reported they were HIV-positive but nearly half (46.0%) did not know their status. In Nigeria, 8.3% reported being HIV-positive while 10.3% did not know their status.

### Antenatal care and substance use

Table 2 summarizes ANC information for the most recent live births in the five years before each interview. Most FSW participants in Kenya (95.6%) reported receiving ANC from a skilled provider (doctor, nurse or midwife) and less than 1% received no ANC. In Nigeria, only 37.5% of FSW received ANC from a skilled provider, while 39.9% did not receive any ANC. In the DRC, 49.0% of FSW received ANC from a skilled provider, while nearly half (45.7%) received no ANC.

**Table 2:** Antenatal care and substance use during pregnancy – most recent live birth in the five years before the survey

Characteristics	DRC *n* (%) (*N* = 247)	Kenya *n* (%) (*N* = 297)	Nigeria *n* (%) (*N* = 283)
Antenatal care provider			
Skilled provider^a^	121 (49.0)	284 (95.6)	106 (37.5)
Unskilled provider^b^	12 (4.9)	8 (2.7)	63 (22.3)
No ANC	113 (45.7)	2 (0.7)	113 (39.9)
Don’t know/Declined to answer	1 (0.4)	3 (1.0)	0
4 + visits	67 (27.1)	227 (76.4)	78 (27.6)
Took iron (any)	104 (42.1)	271 (91.2)	90 (31.8)
Took deworming medication	97 (39.3)	216 (73.0)	46 (16.3)
Took IPTp^c^ (at least one dose)	100 (40.5)	168 (85.3)^d^	182 (64.3)
HIV test done	163 (66.0)	285 (96.0)	223 (78.8)
Any alcohol use^e^	190 (76.9)	155 (52.4)	202 (71.4)
Any drug use	152 (61.5)	86 (29.1)	55 (19.4)

^a^ Doctor, nurse, midwife, or aux midwife

^b^ Traditional birth attendant, community health worker, other

^c^ Intermittent preventive treatment during pregnancy for malaria

^d^ Excludes Nairobi where IPTp for malaria is not indicated

^e^ Self-reported

Most women in Kenya reported four or more ANC visits (76.4%). The percentages were much lower in the DRC (27.1%) and Nigeria (27.6%). Most participants in Kenya (91.2%) reported taking at least some iron supplements during pregnancy, but this was true for only 42.1% in the DRC and 31.8% in Nigeria. Similarly, deworming medication use was higher in Kenya (73.0%), followed by the DRC (39.3%) and Nigeria (16.3%). In Kenya and Nigeria, a majority took anti-malarial medication at least once during pregnancy (85.3% and 64.3% respectively) while 40.5% took it in the DRC. HIV testing was done for 96% of FSW participants in Kenya, 78.8% in Nigeria and 66.0% in the DRC.

When asked why they did not receive ANC, 97% of participants in Nigeria and DRC cited cost as the primary barrier (data not shown in table). In the DRC, 14% also reported stigma.

#### Substance use during pregnancy

Alcohol use during pregnancy was common in all three countries, reported by 52.4% of FSW participants in Kenya, 71.4% in Nigeria, and 76.9% in the DRC. (Table 2). Drug use was frequent in the DRC (61.5%) but less so in Kenya (29.1%) and Nigeria (19.4%). Textual data analysis found that marijuana was most commonly reported in all countries with muguka/khat (an addictive herbal stimulant) also prevalent in Kenya.

### Care during childbirth

Table 3 presents data for the most recent live births in the five years prior to the interview. Nearly all Kenyan FSW (97%) delivered in a health facility and 93.9% reported receiving care from a skilled birth attendant. In the DRC, 68% of women delivered in a health facility with 72.5% reporting a skilled birth attendant. In Nigeria, 26.1% of FSW delivered in health facilities with 34.6% reporting a skilled birth attendant.

When asked why they did not deliver in a health facility the majority of FSW participants in Nigeria (87%) and the DRC (81%) cited high costs as the primary barrier. In the DRC, 26% of respondents also cited stigma as a reason. In Nigeria, transportation was an issue, albeit a minor one (data not shown in table).

**Table 3:** Place and assistance during childbirth - most recent live birth in the five years before the survey

Characteristics	DRC *n* (%) *N* = 247	Kenya *n* (%) *N* = 297	Nigeria *n* (%) *N* = 283
Place of Delivery			
Health facility	168 (68.0)	288 (97.0)	74 (26.1)
Gov’t hospital / clinic	96 (38.9)	227 (76.4)	50 (17.7)
NGO or private hospital / clinic	72 (29.1)	61 (20.5)	24 (8.5)
Not in health facility	79 (32.0)	9 (3.0)	209 (73.9)
Home / another person’s home	48 (19.4)	9 (3.0)	139 (49.1)
Other^a^	31 (12.6)	0	70 (24.7)
Assistance during delivery			
Skilled birth attendant^b^	179 (72.5)	279 (93.9)	98 (34.6)
Traditional birth attendant	2 (0.8)	3 (1.0)	125 (44.2)
Other^c^	66 (26.7)	11 (3.7)	60 (21.2)
Total with unskilled attendant	68 (27.5)	14 (4.7)	185 (65.4)
Don’t know/Declined to answer	0	4 (1.4)	0

^a^ Includes midwife’s office, informal maternity, street, other

^b^ Doctor, nurse, midwife, aux midwife

^c^ Family/friend, neighbor, other sex worker, trainee nurse, community health worker, alone, other

### Birth outcomes

Table 4 presents live births, stillbirths, and early neonatal deaths reported by FSW in the previous five years. The mean number of live births per woman was 1.24 in Kenya, 1.62 in Nigeria, and 1.67 in the DRC. The proportion of total births that were stillbirths was highest in the DRC (11.3%), followed by Nigeria (8.5%), and Kenya (4.9%). Perinatal mortality, which includes stillbirths and early neonatal deaths (0–6 days), followed a similar pattern: DRC (14.5%), Nigeria (11.3%) and Kenya (7.0%). For comparison with DHS and estimates reported in the literature, Table 4 reports these convenience sample-based rates per 1000 total births.

LBW is reported for the most recent live birth in the five years preceding data collection. Birthweight information was primarily available for births attended by a skilled birth attendant. In Kenya and the DRC, 97.9% and 78.1% of FSW, respectively, knew their infant’s birthweight, compared with 23.6% in Nigeria. Among infants with known birthweight, 17.1% in the DRC and 9.7% in Kenya were classified as LBW. In Nigeria, the number of observed LBW cases was insufficient to report a reliable percentage, largely due to the small proportion of women who knew the birthweight.

**Table 4:** Birth outcomes in the five years preceding the survey

Birth Outcomes	DRC *N* = 250	Kenya *N* = 302	Nigeria *N* = 301
Total live births	417	365	485
Don't know/Declined to answer, n (%)	0	2 (0.7)	2 (0.7)
Mean live births per woman	1.67	1.24	1.62
Total stillbirths	53	19	45
Don’t know/Declined to answer, n (%)	5 (2.0)	12 (4.0)	24 (8.0)
Stillbirth rate per 1000 births^a^ (95% confidence interval)	113 (85-146)	49 (30-75)	85 (63-111)
# early neonatal deaths^b^	15	8	15
Perinatal mortality rate per 1000 births^c^ (95% confidence interval)	145 (115–180)	70 (47–100)	113 (87–144)
Most recent live birth in past five years			
Known birth weight, n (%)	193 (78.1)	290 (97.9)	67 (23.6)
Among those with known weight: Low (< 2.5 kg), n (%)	33 (17.1)	28 (9.7)	5^d^

^a^Births = live births + stillbirths

^b^0-6 days

^c^Stillbirths + early neonatal deaths / 1000 births

^d^*n*=5; percentage not reported because the estimate is considered unreliable

### Comparison with reference group

To aid interpretation, outcomes observed among FSW in this study sample are presented alongside data from urban women in each country, drawn from the most recent population-based surveys [14–16]. Stillbirth rates in this study are also compared with national estimates from the Global Burden of Disease (GBD) Study [18]. Key methodological differences between the FSW study and population-based surveys are summarized in Supplemental Table 1. Given these differences, comparisons are intended to provide public health context and should be interpreted as descriptive rather than inferential. Tables 5 and 6 summarize sociodemographic characteristics, maternal health service utilization, and birth outcomes for FSW participants and the reference groups.

**Table 5:** Sociodemographic characteristics and maternal health service utilization among FSW compared with urban women^a^

Indicator	DRC			Kenya		Nigeria	
	FSW %	DHS 2023–2024 urban women %		FSW %	DHS 2022 urban women %	FSW %	DHS 2024 urban women %
Education level							
No education	20.8		3.4	0.7	8.7	5.6	13.8
Any primary	31.7		10.1	41.7	38.2	32.2	9.8
Any secondary or higher	47.2		86.6	57.6	53.0	61.5	76.3
Antenatal care							
Skilled provider^b^	47.8		91.1	95.6	98.9	37.1	82.9
Unskilled provider	4.0		0.2	2.7	0	22.3	6.4
No ANC	45.7		8.7	0.7	1.1	39.9	10.7
Care during childbirth							
Health facility	68.0		95.1	97.0	97.0	26.1	66.3
Other	31.9		4.9	3.0	3.0	73.9	33.7
Skilled birth attendant^b^							
Yes	72.5		96.6	93.9	97.3	34.6	72.7
No	27.5		3.4	4.7	2.7	65.4	27.3

^a^ FSW estimates are from a non-probability convenience sample; comparisons with urban women are descriptive

^b^ Doctor, nurse, midwife, auxiliary midwife

**Table 6:** Adverse birth outcomes among FSW compared with urban women and GBD national estimates^a^

Indicator	DRC			Kenya		Nigeria	
	FSW %	DHS 2023–2024 urban women %		FSW %	DHS 2022 urban women %	FSW %	DHS 2024 urban women %
LBW							
% with known birth weight	78.1		91.0	97.9	97.3	23.6	43.5
% LBW (< 2.5 kg)^b^	17.1		NA^c^	9.7	6.5	^d^	NA^c^
Stillbirth rate^e^	113		17	49	16	85	19
Perinatal mortality rate^f^	145		35	70	33	113	48
			GBD Study 2021 (national)		GBD Study 2021 (national)		GBD Study 2021 (national)
Stillbirth rate^e^	113		36.6	49	28.5	85	53.6

^a^ FSW estimates are from a non-probability convenience sample; comparisons with urban women and GBD data are descriptive

^b^ Among births with known birthweight

^c^ Not available; DHS reports LBW only when ≥ 90% of births nationally have recorded birthweight; this threshold was not reached in the DRC or Nigeria

^d^ Too few observations to report a reliable percentage (*n* = 5)

^e^ Stillbirths per 1000 births (live births + stillbirths)

^f^ Stillbirths + early neonatal deaths (0–6 days) per 1000 births

FSW participants had substantially lower levels of formal education than urban women in the DRC and slightly lower in Nigeria. Educational attainment was broadly similar between groups in Kenya. Descriptively, utilization of ANC with a skilled provider was lower among FSW than among urban women in the DRC (47.8% vs. 91.1%) and Nigeria (37.1% vs. 82.9%), but comparable in Kenya (95.6% vs. 98.9%). Facility-based delivery followed a similar pattern, with lower utilization among FSW participants in the DRC (68.0% vs. 95.1%) and Nigeria (26.1% vs. 66.3%), and no difference observed in Kenya, (97.0% in both groups). Patterns for skilled birth attendance mirrored those for facility-based delivery.

Compared descriptively with urban women, a smaller proportion of FSW reported their infant’s birthweight in the DRC (78.1% vs. 91.0%) and Nigeria (23.6% vs. 43.5%), reflecting lower rates of skilled birth attendance. Among births with known birthweight, the prevalence of LBW was higher among FSW than urban women in Kenya (9.7% vs. 6.5%). Data from Nigeria were insufficient to report a reliable LBW estimate due to the small proportion of infants with known birthweight. DHS did not report LBW prevalence estimates for either the DRC or Nigeria because the national threshold of 90% of births with recorded birthweight was not reached.

When compared descriptively with national GBD estimates, stillbirth rates observed among FSW participants were substantially higher in all three countries: threefold higher in the DRC (113 vs. 36.6 per 1,000 births), 72% higher in Kenya (49 vs. 28.5), and 59% higher in Nigeria (85 vs. 53.6) [18]. GBD does not provide PMR. Relative to DHS estimates for urban women, PMRs were also substantially elevated among FSW in all three countries: DRC (145 vs. 35), Kenya (70 vs. 33), and Nigeria (113 vs. 48).

## Discussion

This study addresses an important evidence gap regarding maternal health among FSW in LMICs by documenting a high burden of adverse birth outcomes in the DRC, Kenya, and Nigeria, including low birth weight, stillbirth, and perinatal mortality. Across all three settings, stillbirth and perinatal mortality rates observed in this study were substantially higher than those reported for urban women in population-based surveys and national estimates. The burden was greatest in the DRC, where stillbirth rates exceeded national estimates more than threefold. MHS utilization varied widely: FSW participants in Kenya reported high uptake of ANC and facility-based delivery (> 95%) and experienced fewer adverse outcomes than those in the DRC and Nigeria, although rates remained elevated. These findings highlight marked inequities in maternal and newborn health and suggest that improving access to MHS is necessary but not sufficient to address the high burden of adverse outcomes. The quality of care and broader structural and social determinants likely also play critical roles.

### Maternal health services and prevention of adverse birth outcomes

While this study cannot identify specific causes of adverse birth outcomes, existing evidence suggests that multiple factors may contribute to the elevated risks observed. The factors discussed below should therefore be interpreted as plausible contextual influences documented in prior research rather than explanations supported directly by the study data.

Approximately half of all stillbirths occur intrapartum, many of which are preventable with timely access to high-quality care during childbirth [19–21]. The lower stillbirth rate among Kenyan FSW, where facility delivery was nearly universal, is consistent with the importance of intrapartum care. However, the persistence of elevated stillbirth rates among Kenyan FSW compared with national estimates suggests that access to intrapartum care alone is not enough to eliminate disparities in birth outcomes.

Antepartum infections are important and potentially preventable contributors to stillbirths [19]. These conditions may be particularly relevant for FSW, who are at higher risk and often face barriers to timely and consistent antenatal care. Malaria accounts for around 20% of stillbirths in sub-Saharan Africa, while syphilis contributes around 11%. In this study uptake of intermittent preventive treatment during pregnancy for malaria was high in Kenya (85.3%) and lower in Nigeria (64.3%) and the DRC (40.5%). Lower uptake may indicate reduced protection against malaria during pregnancy, a known risk factor for stillbirth and other adverse birth outcomes. Similarly, untreated syphilis infection during pregnancy is associated with adverse outcomes in over half of affected pregnancies, including 21% with stillbirths [22]. Antibiotic treatment, particularly if given before the third trimester, significantly reduces stillbirths [23, 24]. Fewer than 50% of FSW participants in the DRC and Nigeria utilized ANC with a skilled provider, and even fewer had more than one visit. Increasing access to early, high-quality ANC could facilitate increased testing and treatment for these infections.

FSW are also at increased risk of HIV. Data to assess the burden of stillbirths attributable to HIV are scarce but it is estimated to be around 0.7% in sub-Saharan Africa and is higher among those not receiving anti-retroviral therapy [19, 25, 26]. In Kenya 96.0% of FSW were tested during their most recent pregnancy but this was true for fewer women in Nigeria (78.8%) and the DRC (66.0%). Lower rates of testing may reduce opportunities for diagnosis and treatment of HIV during pregnancy, which has been associated with adverse birth outcomes. Notably, 46% of DRC participants reported not knowing their HIV status despite recruitment through organizations providing HIV services to FSW, suggesting persistent gaps in HIV testing uptake, knowledge of status, or engagement with available services.

Emerging and epidemic infections may also contribute to adverse birth outcomes. COVID-19 has been associated with increased stillbirth risk, and the study period overlapped with the pandemic [27, 28]. Mpox, which reached epidemic levels in the DRC in 2022, has also been associated with high rates of stillbirth [29]. Neither COVID-19 nor mpox infection was assessed in this study, and their contribution to the observed outcomes cannot be determined. However, these infections highlight additional potentially preventable risks to maternal and newborn health in settings where FSW may face barriers to care.

No specific prevalence data are available for violence against pregnant women although it is known to be associated with stillbirths [5, 19]. Intimate partner violence, which is more prevalent among FSW than among women in general, may contribute to adverse outcomes through both direct physical effects and indirect pathways such as stress and delayed care-seeking [30].

Perinatal mortality was high across all three countries and was largely driven by stillbirths, consistent with possible misclassification between stillbirths and early neonatal deaths [31, 32]. However, early neonatal mortality was also elevated, although to a lesser extent in Kenya, further supporting the importance of access to skilled birth attendance.

The elevated prevalence of LBW observed among FSW, particularly in the DRC, is consistent with increased exposure to risk factors such as maternal infections, poor nutrition, and stress that have been associated with LBW in previous studies. Many of the same conditions associated with stillbirth are also linked to LBW. Preventive interventions for malaria, syphilis and HIV have been shown to significantly reduce its prevalence, underlining once again the negative effects of lack of access to high-quality ANC [24, 29, 33, 34].

Given the high rate of food insecurity among FSW in these countries it is likely that maternal nutritional deficiencies are also contributing to the high LBW prevalence [35, 36]. Likewise, intimate partner violence is associated with LBW – primarily due to preterm birth [37]. Tobacco use and secondhand smoke exposure are additional established risk factors for LBW [38]. Addressing these risks will require coordinated approaches that combine quality ANC with interventions to improve maternal nutrition and prevent violence.

The lack of birthweight data among Nigerian FSW reflects limited access to skilled birth attendance, as infants were not routinely weighed. This data gap is itself indicative of gaps in maternal and newborn care, where even basic postnatal assessments are not consistently performed.

The high rates of alcohol use during pregnancy observed across all three countries, and the particularly high prevalence of drug use in the DRC, may also contribute to adverse outcomes. Substance use during pregnancy is associated with a range of risks, including LBW, preterm birth, and fetal alcohol syndrome, and has been linked to underlying mental health conditions including depression [39, 40]. Integrating culturally appropriate behavioral health interventions with MHS may therefore be important for improving outcomes. However, substance use among FSW may reflect not only individual behaviors but also coping responses to broader structural vulnerabilities, including poverty, violence, food insecurity, stigma, and unstable living conditions. These same factors may contribute independently to adverse maternal and newborn health outcomes.

There are many other causes of adverse birth outcomes. ANC during pregnancy can contribute to improved maternal and neonatal outcomes if the recommended services are provided. Prior research has shown that eight ANC visits can reduce the risk of stillbirths compared to four visits [7, 41]. Likewise, skilled birth attendance reduces maternal mortality and morbidity and improves perinatal outcomes [8].

In this study, MHS utilization among FSW varied substantially across countries shaped by health policies, stigma, and cost. In the DRC and Nigeria, where ANC coverage and skilled birth attendance were lower, addressing the key barriers identified by participants of cost and stigma, is critical to improving access. Stigma may manifest as fear of disclosure of sex work and anticipated or experienced provider discrimination, which can delay care-seeking, limit disclosure of relevant health information, and reduce care quality. This, in turn, may contribute to delayed recognition and management of pregnancy complications. These findings are consistent with prior research identifying financial and stigma-related barriers as major obstacles to care for FSW globally [42]. Prior work also highlights approaches to mitigate stigma including peer-led sex work organizations, multidisciplinary collaborations, and trauma-informed, person-centered care [43].

At the same time, ensuring quality and increasing frequency of visits is also critical [7, 44]. Even when FSW accessed care, recommended preventive interventions were not consistently delivered. Furthermore, survey-based indicators may not reflect the actual quality or skill level of care [44]. Strengthening both access to and quality of MHS is therefore essential to reducing adverse outcomes.

### The case of Kenya

Study participants from Kenya had higher MHS utilization and lower stillbirth and PMR compared with those in the DRC and Nigeria. Over 95% accessed skilled ANC, and 97% delivered in health facilities. Three-quarters had at least four ANC visits and were more likely to receive key recommended services. This study was not designed to assess the impact of specific policy reforms. Nevertheless, government initiatives—such as voucher programs (2008), elimination of user fees for maternal health services in public facilities (2013), and the Linda Mama program (2017), which subsidized maternal healthcare—may have contributed to greater MHS utilization [45].

In addition, FSW are recognized as a key population in Kenya and are engaged through HIV programs, including dedicated clinics, drop-in centers, and community-based outreach. However, similar HIV-focused services exist in the DRC and Nigeria, and maternal health services are not specifically tailored to FSW in any of the three countries. Engagement with HIV services may nonetheless facilitate broader access to care.

Despite these strengths, adverse birth outcomes among Kenyan FSW remained elevated relative to DHS and GBD reference estimates, indicating persistent disparities even in a relatively strong service environment. As MHS utilization improves, greater attention is needed to early initiation, frequency and quality of ANC services, as well as the quality of intrapartum care provided by skilled birth attendants. However, improvements in service utilization and quality alone are unlikely to fully address these outcomes. The factors underlying this residual disparity could not be determined from the available data but may include differences in quality of care, social determinants of health, and other unmeasured risks.

### Social determinants of health

Health services provide a critical opportunity to improve maternal and infant health outcomes [46]. However, reducing adverse birth outcomes among FSW will likely require interventions that extend beyond biomedical care, as these outcomes are also shaped by social determinants such as income, food insecurity, housing, education, gender inequality, and working conditions. Urban environmental factors, including overcrowding, poor sanitation, and limited access to clean water, may further increase exposure to infectious diseases. Although these factors are likely relevant to the adverse outcomes observed among FSW, this study did not directly assess most of them and therefore cannot determine their relative contribution to the disparities identified. Nevertheless, while effective health services can mitigate some risks, attention to underlying social determinants will likely be essential to reducing persistent inequities.

The Sustainable Development Goals emphasize advancing equity and leaving no one behind in the process of economic, social, and environmental development [47]. To advance health equity, populations most affected by inequities must be explicitly prioritized. The World Health Organization framework for monitoring health inequalities includes occupation as a relevant subgroup criterion [47]. This study identifies sex workers as a particularly vulnerable sub-group with poorer maternal and infant health outcomes, underscoring the need for their inclusion in equity-focused monitoring and interventions.

### Limitations

Constructing a sampling frame for FSW is inherently difficult given their status as a hard-to-reach population. This study used a convenience sample recruited through trusted sex worker organizations. While participants were compensated to encourage wide participation, results from this non-random sample are not generalizable beyond the study population.

The extent and direction of sampling bias are uncertain. Participants may have worked more frequently—possibly indicating greater economic need—or may have been healthier and more able to work. Women recovering from a recent birth may not yet have returned to work, potentially reducing their participation. In addition, eligibility required having a child aged five years or younger, excluding some FSW whose recent pregnancies ended in stillbirth, neonatal death, or child death. These factors may have led to underestimation of stillbirth and perinatal mortality rates. Recruitment through sex worker and service organizations may have underrepresented highly marginalized FSW not connected to these networks and women who died from pregnancy-related causes could not participate leading to possible survivorship bias. Together, these factors may have led to underestimation of barriers to MHS and the burden of adverse maternal and birth outcomes. Social desirability bias may have led to overreporting of ANC utilization and HIV testing and underreporting of substance use.

This study relied on retrospective self-report over a five-year period and did not collect complete pregnancy histories, introducing potential recall bias for stillbirths as well as details such as ANC visits and birthweight. Because complete pregnancy histories were not collected, parity could not be determined and is therefore not reported.

Comparisons with population-based data for urban women provide useful context, but are limited by differences in methodology, recall periods and indicator definitions. DHS included stillbirths in denominators for childbirth location and birth attendant. Excluding these may underestimate disparities, as women with stillbirths are less likely to have used MHS. FSW also differ from urban women in the general population in ways that may influence MHS utilization and birth outcomes, including poverty, violence exposure, education, marital status, and other social and occupational factors. As a result, observed differences should not be interpreted as attributable solely to sex work status. Differences in data collection methods may also have influenced reporting of sensitive outcomes and behaviors. DHS data were collected by trained survey enumerators in household settings, whereas this study used staff from local partner organizations conducting interviews in non-household locations. These differences may have affected reporting of sensitive experiences such as stillbirths, substance use during pregnancy, and health service utilization.

Stillbirths were defined as fetal deaths at ≥ 6 months’ gestation. However, gestational age estimates are based on maternal recall and are imprecise due to limited access to ultrasound. Although DHS and GBD use different thresholds (28 weeks/7 months and 20 weeks, respectively), these are also largely recall-based, making reported thresholds approximate and limiting strict comparability across data sources.

High PMR appears largely driven by stillbirths, suggesting possible misclassification, although early neonatal deaths were also elevated. Stillbirth non-response ranged from 2% (DRC) to 8% (Nigeria), which may contribute to underestimation.

Although confidence intervals were calculated for stillbirth and PMR, the non-probability sampling design limits their interpretation; these intervals reflect sample variability and should not be interpreted as population-representative.

Birthweight data were primarily obtained from facility deliveries. Since cost was the main barrier, the poorest women, at higher risk of LBW, are likely underrepresented, potentially underestimating LBW prevalence in the DRC. In Nigeria, birthweight data were largely unavailable due to low rates of facility delivery and limited newborn weighing, constraining assessment of LBW while also reflecting gaps in access to basic maternal and newborn services.

Timing of the first ANC visit was not analyzed because missing data and concerns regarding the accuracy of self-reported gestational age limited the reliability of this measure.

### Strengths

This multicountry study offers extensive data on MHS utilization including number of ANC visits and quality of care indicators such as provision of preventive treatments for anemia, malaria and helminths, and HIV testing. It includes information rarely reported among FSW such as skilled birth attendance and adverse birth outcomes. The use of questions from the DHS facilitated a comparison with findings for urban women in each country studied.

## Conclusion

This study contributes important new evidence on MHS utilization and adverse birth outcomes among FSW in three sub-Saharan African countries. The findings are concerning: stillbirth and perinatal mortality rates were high across all three countries, and LBW was prevalent where birthweight data were available. Many of these adverse outcomes are preventable through timely, high-quality ANC and skilled birth attendance.

Improving maternal and newborn health outcomes for FSW requires addressing key barriers to care, including cost, stigma, and discrimination, while ensuring the quality of services delivered. Achieving equity will also require addressing the social determinants that shape risk among FSW including poverty, food insecurity, unstable housing and exposure to violence. The findings suggest that FSW experience disproportionate risks of poor maternal and newborn outcomes and should be recognized as a high-risk population requiring targeted interventions. Potential approaches include integrating maternal health and HIV services, expanding peer-navigation and community outreach programs, reducing financial barriers to care, and implementing stigma-reduction strategies within health systems. Future research should validate these findings, clarify causal pathways, and evaluate interventions that improve both access to quality care and the underlying social conditions shaping risk.

## Supplementary Information

Supplementary Material 1.

Supplementary Material 2.

## Data Availability

The raw data supporting the conclusions of this article will be made available by the authors, without undue reservation.

## Abbreviations

ANC: Antenatal care
COVID: 19–coronavirus disease of 2019
DHS: Demographic and Health Survey
DRC: Democratic Republic of the Congo
FSW: Female sex workers
GBD: Global burden of disease
HIV: Human immunodeficiency viruses
LBW: Low birthweight
LMIC: Low–and middle–income countries
MHS: Maternal health services
PMR: Perinatal mortality rate
