# Supplementary material for: Maternal health service utilization and birth outcomes among female sex workers in the Democratic Republic of the Congo, Kenya, and Nigeria: results from a cross-sectional survey
Source: BMC Public Health. 2026 Aug 12;26:3053. doi: 10.1186/s12889-026-28825-9 (PMC13693126; doi:10.1186/s12889-026-28825-9)
Supplement: Supplementary file 1 [file 12889_2026_28825_MOESM1_ESM.docx]

**Rapid Assessment 8 Feb 2022**

NOTE: DNK= “Do Not Know” DTA = “Decline To Answer”

**Interviewer Information**. Before completing the survey with the participant, take a moment to complete the interviewer information so that Global Health Promise can track and validate data in this form accurately.

1. Interviewer Name: ___________________
2. a. Location of the interview City___________________________b. Country_________________

c. Partner NGO________________d. Location of interviews________________________________

3. Date of the interview: Month_____________Day_________________Year: 2022
4. Interview Number: If an interview number was not assigned to you, leave this section blank and proceed to submit form. _________
5. Deleted =>7
6. Deleted

**Background Information**

READ THIS STATEMENT:I am going to ask you a series of questions about you and your family. Are you ready to get started?--> If YES, continue on to question A.--> If NO, resolve any questions or concerns before proceeding.

A. What is your age? _______ or check here if the participant does not wish to answer. DTA______
B. What is the highest level of school you completed? Primary_____ Secondary______

Advanced schooling/degree_____ I did not attend school______	DTA_______	Other(specify):_______

C. What is your marital status? Single/never married ____________________--Widowed_____Divorced ____Separated_____DTA___
D. How many adults live in your household? __________ ­­­­­­
E. How many children under age 18 live in your household? _________
F. What are the ages and gender of the children under age 18 who live with you?

| Gender_____  Age___ | Gender_____  Age___ | Gender_____  Age___ | Gender_____  Age___ | Gender_____  Age___ |
| --- | --- | --- | --- | --- |

G. What are the ages and gender of each child you birthed who does not live with you today?

| Gender______  Age______ | Gender______  Age______ | Gender______  Age______ | Gender______  Age______ | Gender______  Age______ |
| --- | --- | --- | --- | --- |

H. Why does this child/these children not live with you today? Record response exactly or write DTA ________________________________________________________________________________________________________________

**Covid Vaccine Questions**

7. Do you plan onto get the COVID vaccine when it becomes available? Yes____=>8 No____=>8 Maybe/DNK____=>8

Already received one or more doses of the vaccine____ =>8

8. What worries do you have about getting the vaccine? No worries_____________DTA____

Record exact response ____________________________________________________________

**Pregnancies and Family Planning**

READ THIS STATEMENT: Now I am going to ask you a series of questions about your pregnancies and their outcomes over the past 5 years, as well as questions about birth control. You can skip any question you do not wish to answer. Ready to begin? --> If YES, proceed to the questions below. --> If NO, resolve any questions or concerns before proceeding.

9. a. What method of birth control to prevent pregnancies do you use now? Female Sterilization___ IUD____

Injections_____ Birth control pills_____ male condom______ Female Condom______

Do Not Use Any_____ DTA______ Other(specify):_______

9b. What is your preferred method of birth control to prevent pregnancy? Female Sterilization___ IUD____ Injections_____ Birth control pills_____ male condom______ Female Condom______

Do Not prefer any method_____ DTA______ Other(specify):_______

9c. What problems do you have getting your preferred method of birth control? No problems_____

too expensive______Do not know where to get it__________ it is unavailable__________

Other [specify]______________________________________

10. Are you pregnant today? Yes______ => 11 No______ => 12 DNK______ => 12 DTA______ => 12
11. How many months pregnant are you? Response: Number of weeks_____ Number of Months_____ DNK_____ DTA_____ =>12
12. How many total pregnancies have you had in the past 5 years? Pregnancies include any time you were pregnant, regardless of the outcome - this includes live births, still births, miscarriages and abortions.

Response:__________ DNK_____ DTA_____

13. How many total live births have you experienced in the past 5 years? A live birth means that the baby was breathing and crying when they were born.

Response:______________________ DNK_____ DTA_____

14. In the past 5 years, how many stillbirths have you experienced? Stillbirth means you were six months pregnant or more and the baby was born but was never alive. Response:_________DNK___ DTA__

14a. In the past 5 years, how many miscarriages have you experienced? Miscarriage mean you were less than 6 months pregnant and the baby was born but never alive. Response_______DNK___DTA___

**Abortions** Now I am going to ask you a series of questions about abortion. Ready to begin? --> If YES, proceed to the questions below. --> If NO, resolve any questions or concerns before proceeding.

15. In the past 5 years, how many abortions have you had? Response:_______=>16 None_____=>18 DNK_____=>18 DTA___=>18
16. For your most recent abortion, what method did you use?

-Pill___16a. What was the name of the pill?________________ DNK________

-Surgical__ 16b. Where was this done? Quack_____ Govt Hospital____Private Hospital/Clinic_____

NGO Clinic________ Other (specify):_____________________________________________________

Local Herb______ DTA_________ -Other (specify):____________________________________________

17. Within three days following your abortion, did you experience

17a. Infection? Yes____No_____DNK___DTA_______=>17b

17b. Excessive bleeding? Yes____No____DNK___DTA____=>17c

17c. Fever? Yes_____No_____DNK_____DTA_____

**HIV**

Now I am going to ask you a series of questions about your HIV status. Remember, I have not recorded your name so no one can know your response.

18. During your most recent pregnancy, were you tested for HIV?

Yes___=>19	No______=>20	DNK____=>20	DTA_____=>20

19. What was the result of the test?

Negative_____=>22 Positive____=>21 Indeterminate___=>22	DNK__=>22	DTA__=>22

20. What is your HIV status today? Negative_____=>23 Positive______=>21 DNK______=>23 DTA_____=>23
21. What month and year did you receive your first HIV positive test result?

Month____________ Year_______ DNK_____ DTA_____

22. Are you currently taking ARVs or antiretroviral medicine?

Yes_______=>24	No _________=> 23	DNK_______=>23	DTA_______=>23

23. Are you currently taking PrEP? Yes_______ No ________DNK______DTA__________=>24

**Antenatal Care**

Now we are going to talk about your most recent pregnancy that resulted in a live birth and antenatal care you may have received during your pregnancy. A live birth means that the baby breathed or cried, even if the baby was only alive for a few minutes. We are not going to talk about miscarriages, stillbirths or abortions in this section. You can skip any question you do not wish to answer. Ready to begin? --> If YES, proceed to the questions below. --> If NO, resolve any questions or concerns before proceeding.

24. How long did your most recent pregnancy last? Weeks_________________Months_______________DNK____
25. How many drinks of alcohol per day did you have during this pregnancy?

Response:_______________ DNK_____ DTA_____

25a. What kind of alcohol did you drink? Beer____ spirits_____local alcohol/brew_____

Other [specify]___________________________________________________________

26. What drugs did you use during this pregnancy?

Record _______________________________________________________________DNK_____ DTA_____

27. Up to what point did you work during this pregnancy? Months__________ DNK_______DTA_____ Comments:_____________________________________________________________________________
28. Who did you see for antenatal care during this pregnancy? *Mark all that apply: Doctor_____=>29	Nurse/Midwife_____=>29	Auxiliary Midwife______=>29	Traditional Birth Attendant_______=>29	Community Health Worker______=>29

DNK______=>31 DTA______=>31 Other (specify): _________________________________________=>29

Did not Receive Care__=>28a

28a. Why didn’t you receive antenatal care during your last pregnancy? Lack of money_______

Clinic location inconvenient_________ clinic hours inconvenient_______ did not need to_________

Attitude of medical staff___________________ DTA____other (specify)___________________________

**Antenatal Care – Care Received**

29. How many times did you see someone for care? __________

How many months pregnant were you went to your first antenatal care visit: ______

During your last pregnancy, how often did you take

29 a. Iron pills or syrup? Once_____ A few times_____ Entire pregnancy_____ Did not take_____ DNK____

29 b. Intestinal worm medication? Once_____ A few times____ Entire pregnancy____ Did not take____ DNK____DTA____

29c. SP/Fansidar (or other malaria treatment)? Once____ A few times___ Entire pregnancy__ Did not take_____ DNK_____DTA____

**Most Recent Birth and Youngest Child**

Now I am going to ask about your most recent pregnancy with your youngest child who was born alive. You can skip any question you do not wish to answer. Ready to begin? --> If YES, proceed to the questions below. --> If NO, resolve any questions or concerns before proceeding.

30. We are going to talk about your most recent live birth. A live birth means that the baby was crying and breathing when they were born. What is your youngest child’s date of birth? Day________Month_________________Year__________
31. Was your youngest child born alone or with other children? Single birth______ Multiple birth________
32. Where did you give birth? Home_____ =>32a Another person’s home_______=>32a

Midwife’s office________ =>32a Informal maternity home____ =>32a Govt hospital/clinic______ =>33 Private hospital/clinic________=>33 NGO hospital/clinic________=>33 DNK______=>33 DTA_______=>33

32a: Why didn’t you give birth at a hospital? Too far_________ too inconvenient________ too expensive______

No time to get there________no transportation________staff is disrespectful_____ I prefer midwife/TBA_____

Other [record comment]____________________________________________________________________

33. Who assisted in the birth of this child? Doctor_____Nurse/Midwife_____Auxiliary Midwife______

Traditional Birth Attendant_____Community health worker______ family member______ friend/neighbor_______Other sex worker_______Birthed child alone without assistance______ DNK_____DTA_____Other (specify):____________________

34. Did you feel respected by the people who helped you give birth? Yes____=>36 No____=>35 DNK____=>36 DTA____=>36 N/A____=>36
35. Why did you not feel respected? Record exact comment:________________________________________
36. Who checked on you after you gave birth? Doctor_____Nurse/Midwife_____Auxiliary Midwife_____

Traditional Birth Attendant______Community health worker______ family member____

friend/neighbor_______Other sex worker______No one______DNK______DTA_____Other (specify):_______________________________________

37. How much did your baby weigh? Kilos___________DNK_______Baby Not Weighed_______DTA______
38. After you gave birth, how long did you stay in that location?

Hours______ Days______ Weeks_____ Months______

39. When did you return to work after you gave birth? Days_______Weeks_______Months_______
40. Where do you leave this child when you go to work? Home with sibs______Home alone______

Take with me_____ at brothel______At bar______At hotspot_____neighbor_____Friend _______

other sex worker______ relative_____paid childcare______Other (specify):________________________

40a. What do you give your children to make them sleep when you are working?

Record response:

41. What is the sex of your youngest child? Male____ Female_____DTA____
42. Is this child alive today? Yes_______=>43 No_______ =>45 DNK_______ =>45 DTA_______ =>45
43. Does this child live with you today? Yes_____ =>45 No_____ =>45 DTA_____=>45
44. Why does this child not live with you? DTA_____

Record exact response:_________________________________________________

**Breastfeeding**

Now we are going to talk about breastfeeding your youngest child. This is the same child we talked about in the last section about birth. You can skip any question you do not wish to answer. Ready to begin? --> If YES, proceed to the questions below. --> If NO, resolve any questions or concerns before proceeding.

45. Did you breastfeed this child? Yes_____=>46	No______=>47	DTA______=>47
46. How long did you breastfeed this *child as the only food source*?

Days _______Weeks_______Months_______Still breastfeeding today_____DTA____

47. If you did not breastfeed for long or at all, why not? Do not have time____

Do not like breastfeeding______No/little milk production_______HIV Positive_______DTA_______

Other: record comments________________________________________________________________

48. How do you feed this child today? Breastfeed only_____=>50 Breastfeed and food______=>49

Food only_____=>49	DTA____=>50

49. At what age (in months) did you start giving this child food? Months:_________ DTA__

49a. What types of food are you giving this child today? Record exact response:______________________________________________________________________________

**Children’s Health**

Now I am going to ask you a series of questions about the health and immunization status of your youngest living child. You can skip any question you do not wish to answer. Ready to begin?--> If YES, proceed to the questions below.--> If NO, resolve any questions or concerns before proceeding. If available, ask the participant's permission to take a photo of the vaccine card(s) for each child living in their household. To maintain confidentiality, cover the child's name if listed on the vaccine card.

50. Does your youngest child have a vaccine card? Yes______DNK_______DTA_______

No_____ =>50a Why doesn’t he/she have a card?___________________________________________________

51. Has your youngest living child ever received a BCG vaccination against tuberculosis, that is, an injection in the arm or shoulder that usually causes a scar? Yes_______No________DNK_______DTA_____
52. Has your youngest living child ever received a measles vaccination, that is, an injection in the arm to prevent measles? Yes________No_______DNK_______DTA_____
53. Where did your youngest living child receive most of his/her vaccines? Govt hospital/clinic________ Private hospital/clinic____NGO _______pharmacy______DNK_____DTA_____

Other location (specify):_________________

My child does not receive vaccines_____=>53a

53a Why doesn’t he/she receive vaccines? Record________________________________________________________________

54. In the past 12 months has your youngest child taken any iron supplements or nutrient powders?

Yes, both iron and nutrient powder_____Yes, only iron_____ Yes, only nutrient powder_____

No, either_______DNK_______DTA_____

55. In the past 6 months, has your youngest living child been treated for intestinal worms? Yes______No_____DNK_____DTA____
56. In the past 2 weeks, has your youngest child experienced diarrhea? Yes_____=>57 No_____=>59 DNK____=> 59 DTA__=> 59
57. Which of the following did your child receive for diarrhea? Did not receive treatment____

Fluid made from special packet called [local name for ORS Packet]_____________

[Local names for pre-packaged ORS liquids] or other pre-packaged ORS liquid_______

Zinc tablets or syrup____ Govt recommended homemade fluid___________

DNK___DTA___Other(specify):______________________________________

**Deaths of Children Alive at Birth**: Now I am going to ask you about the deaths of your children who were alive at birth but have died. I am not asking these questions to upset you but we need to know how to protect children of sex workers so they do not die. I will not ask you about abortions or miscarriages but about the death of all of your children who were alive at birth, which means a child who was born alive and survived outside of your belly for any period, whether that was 1 minute or 10 years. You can skip any question you do not wish to answer. Ready to begin? --> If YES, proceed to the questions below. --> If NO, resolve any questions or concerns before proceeding

58. How many of your children have died after birth? Response______=> 59

None____=> wrap up	DNK_____=> wrap up	DTA______=>wrap up

Now I would like to ask you a few questions about your child/children who died

59. How old was your [first..second…third…] child who died? INSTRUCTIONS: report for each child

| Child 1  Days_____Months_____Years_____ | Child 2  Days_____Months____Years_____ | Child 3  Days_____Months____Years_____ |  |  |
| --- | --- | --- | --- | --- |

Child 4 Days____________Months______Years__________Child 5 Days_________Months_______Years_____

60. Was your child a boy or a girl?

| Child 1  Boy____Girl___ | Child 2  Boy____Girl___ | Child 3  Boy____Girl___ | Child 4  Boy____Girl___ | Child 5  Boy____Girl___ |
| --- | --- | --- | --- | --- |

61. What is the date of their death? Provide estimate if need be

| Child 1  Day__Month__Year_____ | Child 2  Day__Month__Year_____ | Child 3  Day__Month__Year_____ | Child 4  __Month__Year_____ |  |
| --- | --- | --- | --- | --- |

Child 5 day______Month__________Year___________

62. What was the cause of death? Instructions: if due to accident record if due to auto accident or specific details if other type

| HIV____ Malnutrition____  Drug Overdose_____  House Fire_____  Murder_____ Suicide______  Accident______  Other:__________  DNK_____DTA______ | HIV____ Malnutrition____  Drug Overdose_____  House Fire_____  Murder_____ Suicide______  Accident______  Other:_______  DNK_____DTA______ | HIV____ Malnutrition____  Drug Overdose_____  House Fire_____  Murder_____ Suicide______  Accident______  Other:__________  DNK_____DTA______ | HIV____ Malnutrition____  Drug Overdose_____  House Fire_____  Murder_____ Suicide______  Accident______  Other:_______________DNK_____DTA____ | HIV____ Malnutrition____  Drug Overdose_____  House Fire_____  Murder_____ Suicide______  Accident______  Other:_______  DNK_____DTA______ |
| --- | --- | --- | --- | --- |

63. Record additional details about the death:

| Child 1 | Child 2 | Child 3 | Child 4 | Child 5 |
| --- | --- | --- | --- | --- |

64. Was your child tested for HIV before their death?

| Child 1  Yes____=>65a  No____=>66  DNK___=>67  DTA____=>67 | Child 2  Yes_____=>65a  No____=>66  DNK____=>67  DTA____=>67 | Child 3  Yes_____=>65a  No____=>66  DNK____=>67  DTA____=>67 | Child 4  Yes____=>65a  No____=>66  DNK____=>67  DTA____=>67 | Child 5  Yes____=>65a  No____=>66  DNK____=>67  DTA____=>67 |  |  | Yes__=>65a No__=>66 DNK__=>67, DTA__=>67 | Yes__=>65a No__=>66 DNK__=>67, DTA__=>67 | Yes__=>65a No__=>66 DNK__=>67, DTA__=>67 |
| --- | --- | --- | --- | --- | --- | --- | --- | --- | --- |

65a. What was the result?

| Child 1  Positive___  Negative___  Indeterminate___  DNK____  DTA____ =>67 | Child 2  Positive___  Negative___  Indeterminate___  DNK____  DTA____ =>67 | Child 3  Positive___  Negative___  Indeterminate___  DNK____  DTA____ =>67 | Child 4  Positive___  Negative___  Indeterminate___  DNK____  DTA____ =>67 | Child 5  Positive___  Negative___  Indeterminate___  DNK____  DTA____ =>67 |
| --- | --- | --- | --- | --- |

66. Why wasn’t your child tested for HIV?

| Child 1  I didn’t think it was necessary___  Too expensive___  Not convenient____  Other____record response | Child 2  I didn’t think it was necessary___  Too expensive___  Not convenient____  Other____record response | Child 3  I didn’t think it was necessary___  Too expensive___  Not convenient____  Other____record response | Child 4  I didn’t think it was necessary___  Too expensive___  Not convenient____  Other____record response | Child 5  I didn’t think it was necessary___  Too expensive___  Not convenient____  Other____record response |
| --- | --- | --- | --- | --- |

**Go to wrap up=>**

**Wrap Up**

67. Is there anything else you wish to share about your experiences as a mother and sex worker?

Record exact Response:________________________________________________________________DTA:____

68. This is our final question! What can organizations like [NGO name] do to help you and your children during the pandemic? DTA___

Record exact response:_________________________________________________________________________
