# Supplementary material for: Maternal health service utilization and birth outcomes among female sex workers in the Democratic Republic of the Congo, Kenya, and Nigeria: results from a cross-sectional survey
Source: BMC Public Health. 2026 Aug 12;26:3053. doi: 10.1186/s12889-026-28825-9 (PMC13693126; doi:10.1186/s12889-026-28825-9)
Supplement: Supplementary file 2 [file 12889_2026_28825_MOESM2_ESM.docx]

**Supplemental Table 1.** Key differences between FSW study and Demographic and Health Surveys (DHS) used for comparison.

| **Variable** | **FSW Study 2022-2023** | **DRC DHS 2023-2024** | **Kenya DHS**  **2022** | **Nigeria DHS 2024** |
| --- | --- | --- | --- | --- |
| Pregnancy history | Number of live births, stillbirths, abortions and miscarriages in previous 5 years | Full pregnancy history | Full pregnancy history | Full pregnancy history |
| Antenatal care | Most recent live birth  Last 5 years | Most recent birth – live or still  Last 2 years | Most recent birth  – live or still  Last 2 years | Most recent birth  – live or still  Last 2 years |
| Place and attendant at childbirth | Most recent live birth  Last 5 years | All births – live and/or still  Last 2 years | All births – live and/or still  Last 2 years | All births – live and/or still  Last 2 years |
| Stillbirth definition | 6 months gestation | 28 wks / 7 mos gestation | 28 wks / 7 mos gestation | 28 wks / 7 mos gestation |
| Stillbirth rate | Stillbirths  Last 5 years | Stillbirths  Last 5 years | Stillbirths  Last 5 years | Stillbirths  Last 5 years |
| Perinatal mortality rate | Stillbirths + early neonatal deaths^a^ Last 5 years | Stillbirths + early neonatal deaths^a^ Last 5 years | Stillbirths + early neonatal deaths^a^ Last 5 years | Stillbirths + early neonatal deaths^a^ Last 5 years |
| Low birth weight | Most recent live birth with known BW^b^ last 5 years | NA^c^ | All live births with known BW last 2 years | NA^c^ |

^a^ 0-6 days

^b^ BW: birth weight

^c^ Not available; DHS reports low birth weight only when ≥90% of births nationally have recorded birthweight; this threshold was not reached in the DRC or Nigeria
